# TET2 downregulation limits oxidative stress-induced cytotoxicity of high dose ascorbate in dabrafenib-resistant melanoma cells

**DOI:** 10.20517/cdr.2026.42

**Published:** 2026-08-03

**Authors:** Eleicy Nathaly Mendoza, Serena Castelli, Angela De Cristofaro, Enrico Desideri, Grazia Graziani, Pedro Miguel Lacal, Maria Rosa Ciriolo, Fabio Ciccarone

**Affiliations:** ^1^Department of Biology, University of Rome Tor Vergata, Rome 00157, Italy.; ^2^Department for the Promotion of Human Science and Quality of Life, San Raffaele Open University, Rome 00166, Italy.; ^3^IRCCS San Raffaele Roma, Rome 00166, Italy.; ^4^Department of Life Sciences, Health and Health Professions, Link Campus University, Rome 00165, Italy.; ^5^Department of Systems Medicine, University of Rome Tor Vergata, Rome 00133, Italy.; ^6^Laboratory of Molecular Oncology, IDI-IRCCS, Rome 00167, Italy.

**Keywords:** BRAF inhibitors, 5hmC, oxidative stress, drug resistance

## Abstract

**Aim:** This study investigates the role of Ten-Eleven Translocation (TET) dioxygenases in melanoma-acquired resistance to BRAF inhibitors (BRAFi), focusing on their contributions to epigenetic remodeling and redox balance.

**Methods:** Dabrafenib-resistant A375 and M14 melanoma cell lines were generated and analyzed for TET family expression. Transcriptomic data from independent RNA-seq datasets of BRAFi-resistant melanoma models were used for validation. Cells were treated with high-dose L-ascorbic acid (ASC), a cofactor enhancing TET activity, and assessed for 5-hydroxymethylcytosine (5hmC) levels, reactive oxygen species (ROS) production, and cytotoxicity. Antioxidant capacity and expression of GLUT1, a transporter for oxidized ASC, were evaluated. TET2 was silenced to determine its functional role in ASC-mediated effects.

**Results:** BRAFi-resistant cells displayed reduced TET2 expression and altered levels of other TET family members, consistent with RNA-seq data. ASC treatment increased 5hmC levels and induced ROS-dependent cytotoxicity predominantly in parental cells, while resistant cells were less sensitive. Resistant cells exhibited enhanced antioxidant defenses and reduced GLUT1 expression. TET2 silencing diminished ASC-induced 5hmC accumulation, ROS production, and GLUT1 expression, indicating a central role for TET2 in mediating ASC cytotoxicity.

**Conclusion:** These findings identify a TET2-dependent epigenetic–metabolic axis that regulates redox vulnerability in melanoma. The link between TET2 activity, GLUT1 expression, and ROS-mediated cytotoxicity highlights potential biomarkers and therapeutic targets to overcome resistance to BRAFi.

## INTRODUCTION

The advent of targeted therapies against the mitogen-activated protein kinase (MAPK) pathway - most notably BRAF inhibitors (BRAFi) (vemurafenib, dabrafenib, and encorafenib) and MEK inhibitors - has transformed the management of BRAF V600-mutated metastatic melanoma. Nevertheless, durable clinical responses remain uncommon because resistance almost invariably emerges^[1,2]^. Contemporary evidence indicates that this refractoriness arises via both genetic alterations and non-genetic mechanisms, including epigenetic remodeling and phenotype plasticity that enable rapid adaptation under drug pressure^[2,3]^.

Mechanistically, epigenetic reprogramming - encompassing DNA methylation changes, histone modifications, and noncoding RNAs - cooperates with canonical resistance alterations to either reactivate MAPK signaling or divert signaling toward alternative pro-survival pathways^[4]^. In parallel, epigenetically guided phenotype switching from a proliferative, differentiated state to a more invasive, dedifferentiated state fosters drug tolerance and disease progression^[5]^. These processes, documented across clinical specimens and preclinical models of BRAFi resistance, underscore the contribution of reversible, epigenetically encoded states to treatment failure^[4]^.

Within this epigenetic framework, particular attention has focused on the Ten-Eleven Translocation (TET) family of DNA hydroxylases - TET1, TET2, and TET3 - which catalyze the stepwise oxidation of 5-methylcytosine (5mC) to 5-hydroxymethylcytosine (5hmC) and its further oxidized derivatives (5fC, 5caC), thereby governing active DNA demethylation dynamics^[6]^. Since the identification of 5hmC in mammalian genomes as a distinct epigenetic mark, TET-mediated cytosine oxidation has been linked to the regulation of gene expression programs relevant to lineage identity, development, and cancer^[7]^. Although no specific TET enzyme modulators are currently under clinical investigation, several compounds already approved as anticancer agents have been shown to affect TET activity or expression^[8]^. This is the case for ASC, which acts as a cofactor for Fe^2+^/2-oxoglutarate–dependent dioxygenases, including TET proteins^[9]^. Notably, high-dose ASC can reach pharmacological plasma concentrations capable of exerting therapeutic effects in cancer through both epigenetic modulation and the induction of oxidative stress^[10]^.

Melanoma is characterized by a striking global loss of 5hmC, a feature that distinguishes malignant lesions from benign melanocytic counterparts and correlates with aggressive biological behavior^[11]^. This 5hmC depletion, widely replicated across cohorts, provides one of the most robust epigenetic signatures of melanoma and implies impaired TET activity along tumor evolution^[12]^. Functional genetics reinforces this view: *in vivo* ablation of Tet2 accelerates NRAS-driven melanomagenesis and progression, consistent with a tumor-constraining role for TET2 within the melanocyte lineage^[13,14]^. Collectively, these data position the TET–5hmC axis as an integral element of melanoma biology with potential implications for therapy response.

Despite this strong biological rationale, a direct causative role for TET proteins in resistance to BRAFi has not been established. The present work is designed to address this gap by examining the relationship between TET enzymes and dabrafenib resistance in melanoma cells. We aim to provide a foundation for evaluating whether these epigenetic factors may represent potential targets to eradicate resistant cells or enhance the efficacy of current therapies.

## METHODS

### Reagents and chemicals

Dabrafenib (HY-14660) and L-ascorbic acid 2-phosphate (p-ASC) (HY-103701) were obtained from MedChemExpress, NJ, USA. L-ascorbic acid (ASC) (A4403), formalin solution neutral buffered (HT5012), Methyl cellulose (M0512), N-acetylcysteine (NAC) (A7250), and Trolox (648471) were obtained from Sigma-Aldrich, St. Louis, MO, USA. Stock solutions of ASC, p-ascorbate, Trolox, and NAC were prepared in phosphate-buffered saline (PBS). The 0.4% Trypan Blue solution (17-942E) was from Lonza, Basel, Switzerland. 2′,7′-Dichlorofluorescein diacetate (DCFDA; D0063) was from Chemodex, St. Gallen, Switzerland. Goat anti-mouse (172-1011) and anti-rabbit (172-1019) IgG (H + L)-horseradish peroxidase-conjugated, Tris-based (1610716) and sodium dodecyl sulfate (SDS) (161-0300) were from BioRad Laboratories, Hercules, CA, USA. JC-1 (420200) is from Calbiochem, San Diego, CA, USA. Dabrafenib was dissolved in dimethyl sulfoxide (DMSO) to prepare a 10 mM stock solution, which was stored at -20 °C.

### Cell lines and culture conditions

The human melanoma cell line A375 was obtained from the European Collection of Cell Cultures (catalog n. 88113005, Salisbury, UK), whereas the human melanoma cell line M14 was kindly provided by Dr. Zupi G. “Istituto Regina Elena” (Rome, Italy). Chemoresistant A375-R and M14-R were generated in our laboratories by exposing the parental cell line to increasing concentrations of dabrafenib (up to 1 µmol/L) for 4 months and validated according to our previous works^[15,16]^. All cells were authenticated via short tandem repeat (STR) profiling (BMR Genomics, Padova, Italy). The results exhibited an allele percent match (EV value) of 89%-100% against the reference database. Consequently, in accordance with the International Cell Line Authentication Committee (ICLAC) guidelines, these cell lines were considered authenticated. Cells were maintained in Roswell Park Memorial Institute (RPMI) 1640 medium supplemented with 10% fetal bovine serum, 2 mM L-glutamine, 100 U/mL penicillin–streptomycin in a humidified atmosphere containing 5% CO_2_ at 37 °C. A375-R cells and M14-R were continuously cultured in the presence of 1 µM dabrafenib, which was refreshed every 72 h at each passage. All cell lines were confirmed to be mycoplasma-free (MycoAlert® Mycoplasma Detection Kit, Lonza, Basel, Switzerland) before cryopreservation and used between passages 5 and 15 for all experiments.

### Gene silencing by siRNA

*TET2* expression was transiently silenced using a *TET2*-specific small interfering RNA (code SASI_HS02_00328773, Merck Life Science) and Lipofectamine™ 3000 Transfection Reagent (Thermo Fisher Scientific), following the manufacturer’s instructions adapted for a 6-well plate format. Scramble negative control was obtained using MISSION® siRNA Universal Negative Control #1 (code SIC001, Merck Life Science). Cells were seeded 24 h prior to transfection to achieve approximately 60%-70% confluence. For each well of a 6-well plate, 75 pmol of *TET2 or SCR* siRNA were complexed with 7.5 µL of Lipofectamine™ 3000 in Opti-MEM™ Medium according to the manufacturer’s recommendations for siRNA transfection. The transfection complexes were added to the cells, which were incubated for 24 h under standard culture conditions before further analyses or treatments. The efficiency of TET2 knockdown was confirmed by Western blot analysis.

### Trypan blue exclusion assay

Cell viability was assessed using the Trypan blue exclusion assay. After treatment, equal volumes of cell suspension and 0.2% (v/v) Trypan blue solution were mixed. Viable cells remained unstained, whereas non-viable cells appeared blue. Cells were counted manually using a Thoma cell counting chamber.

### MTT assay

Cells were seeded in 96-well plates and treated with the indicated compounds for the specified times. After treatment, the MTT (3-[4,5-dimethylthiazol-2-yl]-2,5 diphenyl tetrazolium bromide) reagent was added to each well and incubated for 4 h at 37 °C to allow formation of formazan crystals. The medium was then removed, and the crystals were dissolved in DMSO. Absorbance was measured at 570 nm using a microplate reader (TECAN Infinite 200 pro), and cell viability was expressed as a percentage relative to untreated controls. To evaluate the influence of glucose availability on ASC-induced cytotoxicity, cells were cultured in RPMI-1640 medium supplemented with glucose at increasing concentrations (2.5, 5, 10, and 20 mM) and subsequently treated with ASC. To assess the involvement of oxidative stress, cells were pretreated with NAC (5 mM) for 4 h prior to ASC exposure, whereas Trolox (50 µM) was administered simultaneously with ASC as a co-treatment. For 2D culture experiments, cells were treated with 0.5 or 1 mM ASC or p-ASC for 72 h. For three-dimensional (3D) spheroid cultures, cells were treated with 0.5, 1, or 2 mM ASC for 5 days.

### 3D spheroid formation

3D spheroids were generated using 96-well U-bottom plates. Cells were suspended in RPMI-1640 medium (100 µL) supplemented with methylcellulose at a final concentration of 10 mg/mL, obtained by diluting a 100 mg/mL methylcellulose stock solution 1:10 in culture medium. Cells were seeded at a density of 2,000 cells per well and centrifuged for 10 min at 400 × *g* to promote spheroid formation. Spheroid development was monitored after 24 h using an inverted light microscope (Axio Observer microscope, Zeiss). Once spheroids were formed, treatments were applied (0.5, 1, or 2 mM ASC for 5 days). Cell viability was evaluated using the MTT assay according to the manufacturer’s instructions.

### Intracellular reactive oxygen species detection

Cells were seeded at a density of 8 × 10^4^ cells/mL in 24-well plates and treated with 0.5 mM ASC or p-ASC for 24 h before the assay. Following treatment, the culture medium was removed and replaced with fresh medium (without FBS) containing 20 µM DCFDA. Cells were incubated for 30 min at 37 °C in the dark. Cells were then washed three times with PBS, and fluorescence was observed with 485 nm excitation and 535 nm emission using a fluorescence microscope (Zeiss Axio Observer).

### JC-1 assay

Mitochondrial membrane potential was assessed using the JC-1 probe, following the same procedure used for reactive oxygen species (ROS) detection. Cells were seeded at a density of 8 × 10^4^ cells/mL in 24-well plates. After 24 h, cells were treated with 0.5 mM ASC or p-ASC for 24 h. Following treatment, the culture medium was removed and replaced with fresh medium (without FBS and Phenol Red) containing 2.5 μM JC-1. Cells were incubated for 30 min at 37 °C in the dark and then washed three times with PBS. Green (monomeric) and red (J-aggregate) fluorescence were acquired separately using a fluorescence microscope (Zeiss Axio Observer), and the red-to-green fluorescence ratio was used as an index of mitochondrial membrane potential.

### Intracellular glutathione quantification (GSH/GSSG assay)

Intracellular glutathione levels were measured using the GSH/GSSG-Glo™ Assay (Promega, Madison, WI, USA) according to the manufacturer’s instructions, with minor modifications for adherent cells. After treatment with 0.5 mM ASC for 24 h, the culture medium was removed, and cells were lysed by adding 50 µL per well of either Total Glutathione Lysis Reagent or Oxidized Glutathione Lysis Reagent. Plates were shaken for 5 min at room temperature to ensure complete lysis. Subsequently, 50 µL of Luciferin Generation Reagent was added, and plates were incubated for 30 min at room temperature. Then, 100 µL of Luciferin Detection Reagent was added, followed by a 15 min incubation, and luminescence was measured using a microplate luminometer. Background luminescence from wells without cells was subtracted from all measurements to obtain net relative luminescence units (RLU). The GSH/GSSG ratio was calculated using the manufacturer’s instructions.

### Western blot analysis

Cell pellets were resuspended in lysis buffer (50 mM Tris-HCl pH 7.4, 150 mM NaCl, 1 mM EDTA, 1% Triton™ X-100, 0.5% sodium deoxycholate, 0.1% SDS, 10 mM sodium fluoride, 5 mM sodium pyrophosphate, and 2 mM sodium orthovanadate) supplemented with a protease inhibitor cocktail (AMRESCO, M221). Homogenates were sonicated, and protein concentrations were determined using the Lowry method. Protein lysates were mixed with sample buffer (0.125 M Tris-HCl pH 6.8, 4% SDS, 20% glycerol, 10% β-mercaptoethanol, and 0.004% bromophenol blue), separated by sodium dodecyl sulfate–polyacrylamide gel electrophoresis (SDS-PAGE), and transferred onto nitrocellulose membranes (Bio-Rad). Membranes were incubated with the following primary antibodies (1:1,000 dilution): TET1, TET2, TET3, GLUT1, CAT, SOD2, α-actinin, and β-actin from Cell Signaling Technology. For normalization of Western blotting analysis, α-actinin and β-actin were used as loading controls for proteins resolved on 6.5% and 10%-12% polyacrylamide gels, respectively. To ensure accurate data interpretation, the corresponding loading control is shown alongside each target protein, including when shared within the same panel. Secondary antibodies used were goat anti-rabbit IgG (H + L)–HRP conjugate and goat anti-mouse IgG (H + L)–HRP conjugate from Bio-Rad. Signals were detected using Clarity Max Western ECL Substrate (Bio-Rad) and acquired using a FluorChem imaging system (Alpha Innotech). Densitometric analysis was performed using ImageJ software.

### Immunofluorescence analysis of 5hmC

Cells were seeded at a density of 8 × 10^4^ cells/ml on glass coverslips or glass-bottom dishes. At the end of the treatment with 0.5 mM ASC or p-ASC for 24 h, the culture medium was removed, and cells were washed with PBS before fixation. Cells were fixed with formalin solution for 15 min at room temperature and washed three times with PBS. Cells were permeabilized with 0.1% Triton X-100 for 10 min and incubated with RNase for 2 h at 37 °C. For detection of 5-hmC, DNA was denatured by incubating cells with 4 N HCl for 15 min at room temperature, followed by neutralization with 100 mM Tris-HCl (pH 8.5) for 10 min. Cells were then washed with PBS and blocked with 3% goat serum in PBST for 1 h at room temperature. Samples were incubated overnight at 4 °C with rabbit anti-5-hydroxymethylcytosine (5hmC; Active Motif) primary antibody diluted in blocking buffer. After washing with PBST, cells were incubated for 1 h at room temperature with the appropriate Alexa Fluor–conjugated secondary antibodies. Nuclei were counterstained with 4′,6-diamidino-2-phenylindole (DAPI) for 10 min. Coverslips were washed with PBS and mounted on glass slides. Fluorescence images were acquired using a confocal fluorescence microscope (Olympus) and quantified with ImageJ software.

### Bioinformatic analyses

Three independent RNA-seq analyses from publicly available datasets were assessed. First, RNA-seq data from vemurafenib-sensitive and -resistant melanoma cell lines were compared and are available in the GSE203545 dataset. Resistant models were generated through chronic drug exposure as described in the original study^[17]^. Samples were grouped according to cell line status (parental *vs.* vemurafenib-resistant), and only cell line pairs with detectable baseline TET expression in the parental (sensitive) cells were included, while those with negligible expression were excluded to ensure meaningful comparative analyses. Gene expression levels were quantified as fragments per kilobase of transcript per million mapped reads (FPKM), normalizing read counts for both gene length and sequencing. Statistical analysis was performed by a paired *t*-test because each vemurafenib-resistant melanoma cell line was generated from its corresponding parental cell line. Thus, parental and resistant samples constitute matched pairs rather than independent groups, making a paired analysis the most appropriate approach to assess resistance-associated changes in TET gene expression while accounting for baseline differences among cell lines. Second, gene expression modulation was analyzed in the human metastatic melanoma cell line WM239A overexpressing BRAFV600E using the publicly available dataset GSE117123. In this dataset, cells were treated with the BRAFi encorafenib (LGX818) over a time course ranging from one to three weeks. Prior to analysis, genes with fewer than 60 total reads were excluded. The remaining genes were analyzed using the DESeq2 package, which was used to normalize read counts for library size and estimate gene-wise dispersion parameters. Normalized data were subsequently used for downstream differential TET gene expression analyses, and statistical significance was determined relative to untreated BRAFV600E controls. Finally, to assess correlations between genes of interest in a large cohort of melanoma patients, Spearman’s correlation analysis was performed using the Skin Cutaneous Melanoma (SKCM) dataset from The Cancer Genome Atlas (TCGA), accessed via the GEPIA2 website^[18]^. This cohort includes RNA-seq data from over 400 melanoma tumor samples, processed through a standardized pipeline and normalized as Transcripts Per Million (TPM). All available tumor samples were included in the analysis without additional exclusion criteria.

### Statistical analysis

All experiments were performed in *n* = 3 independent biological replicates. Data are presented as the mean ± standard deviation (SD). Statistical analyses for multiple comparisons were performed by Graphpad Prism 9 using one-way analysis of variance (ANOVA) followed by Tukey’s post-hoc test, while two-tailed Student’s *t*-tests were applied when comparing two groups. A *P*-value < 0.05 was considered statistically significant.

## RESULTS

### TET expression levels are altered in melanoma cells resistant to BRAFi

**Figure 1:**
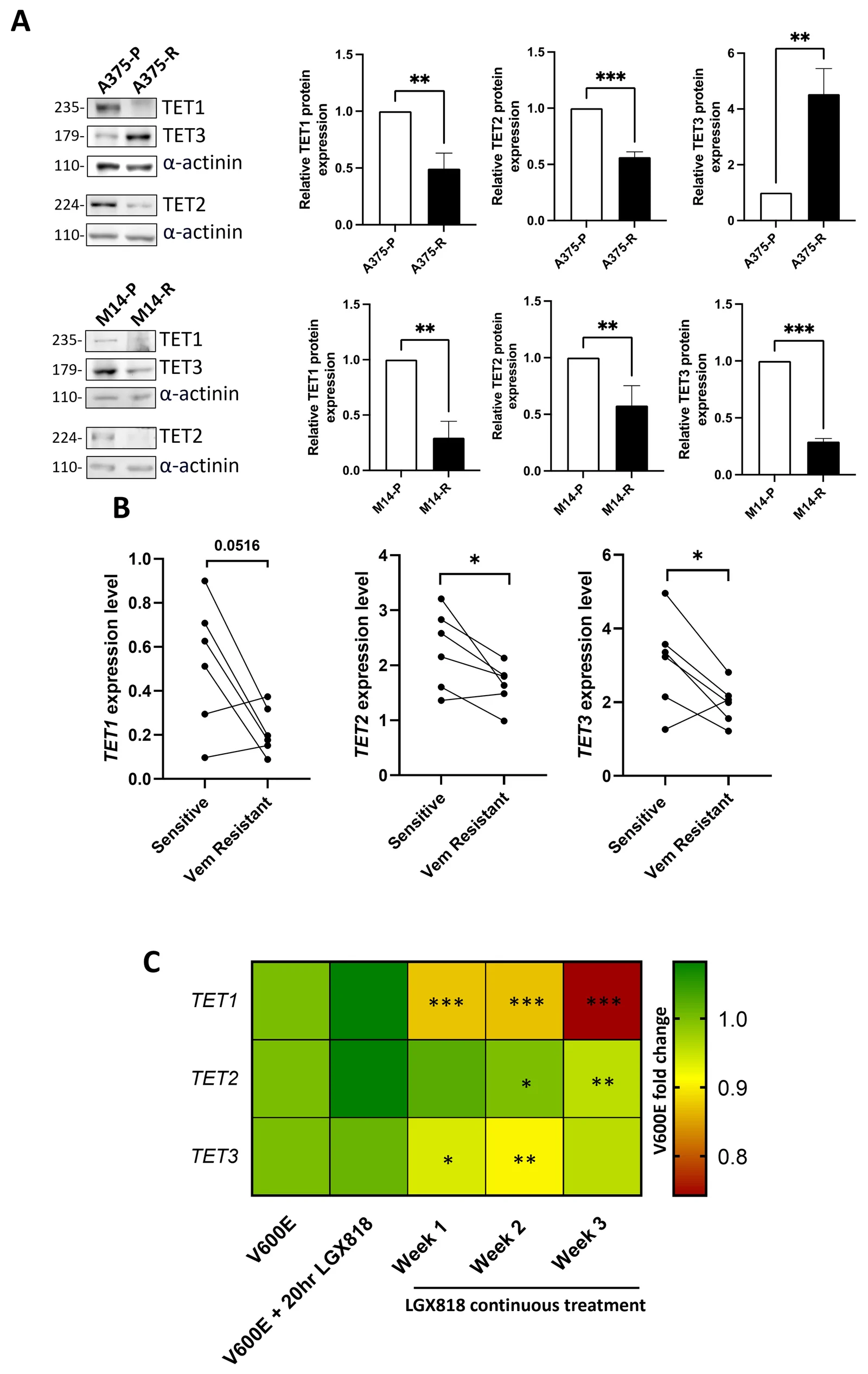
Analysis of TET expression levels in melanoma cells resistant to BRAFi. (A) Western blot of TET proteins in A375 and M14 dabrafenib-sensitive and -resistant cells; images are representative of three independent western blots that were quantified and shown in densitometric analysis. *n* = 3 independent biological replicates were performed. Statistical analysis was performed by unpaired *t*-test; (B) Expression levels of *TET* genes from RNA-seq of different melanoma cell lines resistant to vemurafenib; statistical analysis was performed by paired *t*-test; (C) Expression levels of *TET* genes from RNA-seq of a melanoma cell line overexpressing BRAFV600E and treated with encorafenib/LGX818; statistical significance was referred to V600E-overexpressing cells and assessed by one-way ANOVA. ^*^P < 0.05; ^**^P < 0.01; ^***^P < 0.001. TET: Ten-Eleven Translocation; BRAFi: BRAF inhibitors; ANOVA: analysis of variance.

To investigate the contribution of TET hydroxylases to BRAFi resistance, we generated A375 and M14 mutated BRAF(V600E) melanoma cell lines with acquired resistance to dabrafenib (hereafter referred to as A375-R and M14-R) to compare the abundance of TET1, TET2, and TET3 proteins with their parental counterparts (A375-P and M14-P). Immunoblot analysis revealed an overall reduction in TET protein levels in both resistant cell lines, with the exception of TET3, which was upregulated in A375-R cells [Figure 1A]. Analysis of publicly available RNA-seq datasets demonstrated that *TET2* and *TET3* genes were significantly downregulated in response to other BRAFi, as observed in melanoma cell lines resistant to vemurafenib [Figure 1B] or treated with encorafenib (LGX818) [Figure 1C]. Collectively, these findings support a role for TET enzymes in the adaptive response to BRAFi therapy.

**Figure 2:**
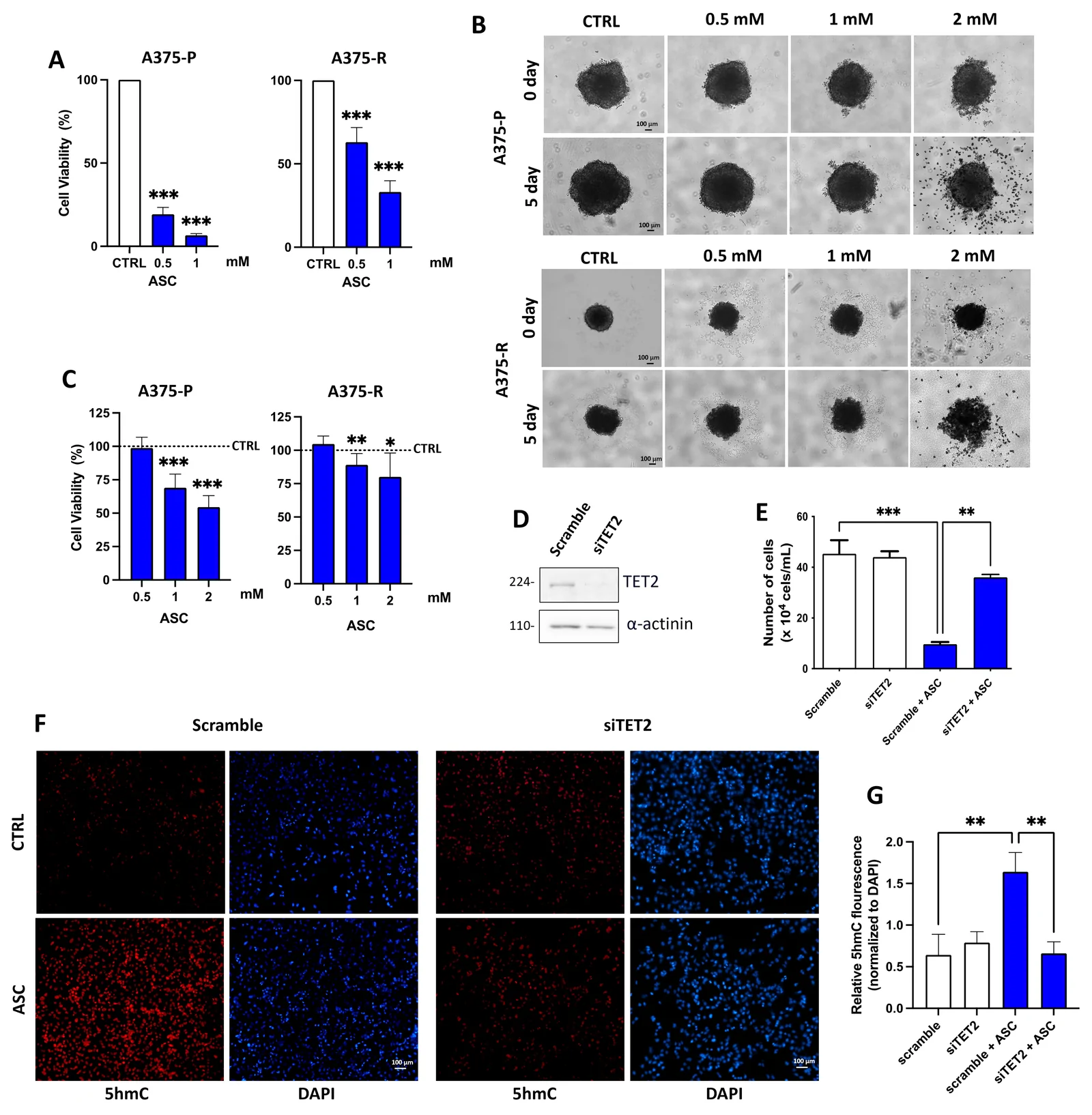
Effect of ASC treatment on cell viability and 5hmC levels in A375 cells. (A) MTT assay of A375-P and A375-R treated with 0.5 and 1 mM ASC; (B) Representative images and (C) MTT assay of 3D cultures of A375-P and A375-R treated with 0.5, 1, and 2 mM ASC. Scale bar = 100 µm. The dashed line represents the untreated CTRL baseline (100%); (D) Western blot showing *TET2* silencing in A375-R cells; images are representative of three independent western blots; (E) Direct cell counting of A375-R cells silenced for *TET2* with/without 0.5 mM ASC; (F) Fluorescence quantification and (G) representative images of 5hmC levels in A375-R cells silenced for *TET2* with/without 0.5 mM ASC. Scale bar = 100 µm. Where indicated, CTRL refers to PBS used as the vehicle control for ASC. Statistical analysis in all bar graphs was performed by one-way ANOVA. ^*^P < 0.05; ^**^P < 0.01; ^***^P < 0.001. All experiments were performed in *n* = 3 independent biological replicates. ASC: L-ascorbic acid; 5hmC: 5-hydroxymethylcytosine; MTT: 3-[4,5-dimethylthiazol-2-yl]-2,5 diphenyl tetrazolium bromide; 3D: three-dimensional; TET: Ten-Eleven Translocation; PBS: phosphate-buffered saline; ANOVA: analysis of variance; DAPI: 4′,6-diamidino-2-phenylindole.

ASC is a well-established activator of TET catalytic activity^[19]^ and, when administered at pharmacological doses, exhibits antineoplastic effects^[10]^. Importantly, ASC-driven 5hmC accumulation has been shown to rely predominantly on TET2^[20]^. Based on this evidence, we investigated how the altered TET expression profile in dabrafenib-resistant A375 cells influences their cytotoxic response to ASC, focusing on the specific role of TET2. Cell viability assays performed after 72 h of exposure to 0.5 or 1 mM ASC demonstrated that A375-R cells were less sensitive to ASC compared with parental A375-P cells [Figure 2A]. This differential sensitivity was confirmed in a 3D culture system by measurement of spheroid viability, where resistant cells retained higher viability following ASC treatment, whereas parental cells exhibited a marked reduction in viability, reaching a 50% decrease at 2 mM ASC compared with the untreated control (CTRL) group [Figure 2B and C]. We then silenced TET2 [Figure 2D] and found that ASC no longer reduced cell viability [Figure 2E]. Consistently, immunofluorescence analyses revealed a marked increase in 5hmC upon ASC treatment in scrambled siRNA–transfected cells (scramble), whereas only minimal 5hmC staining was detected after TET2 knockdown (siTET2) [Figure 2F and G].

### ASC treatment promotes cell death in A375 melanoma cells in a ROS-dependent manner

**Figure 3:**
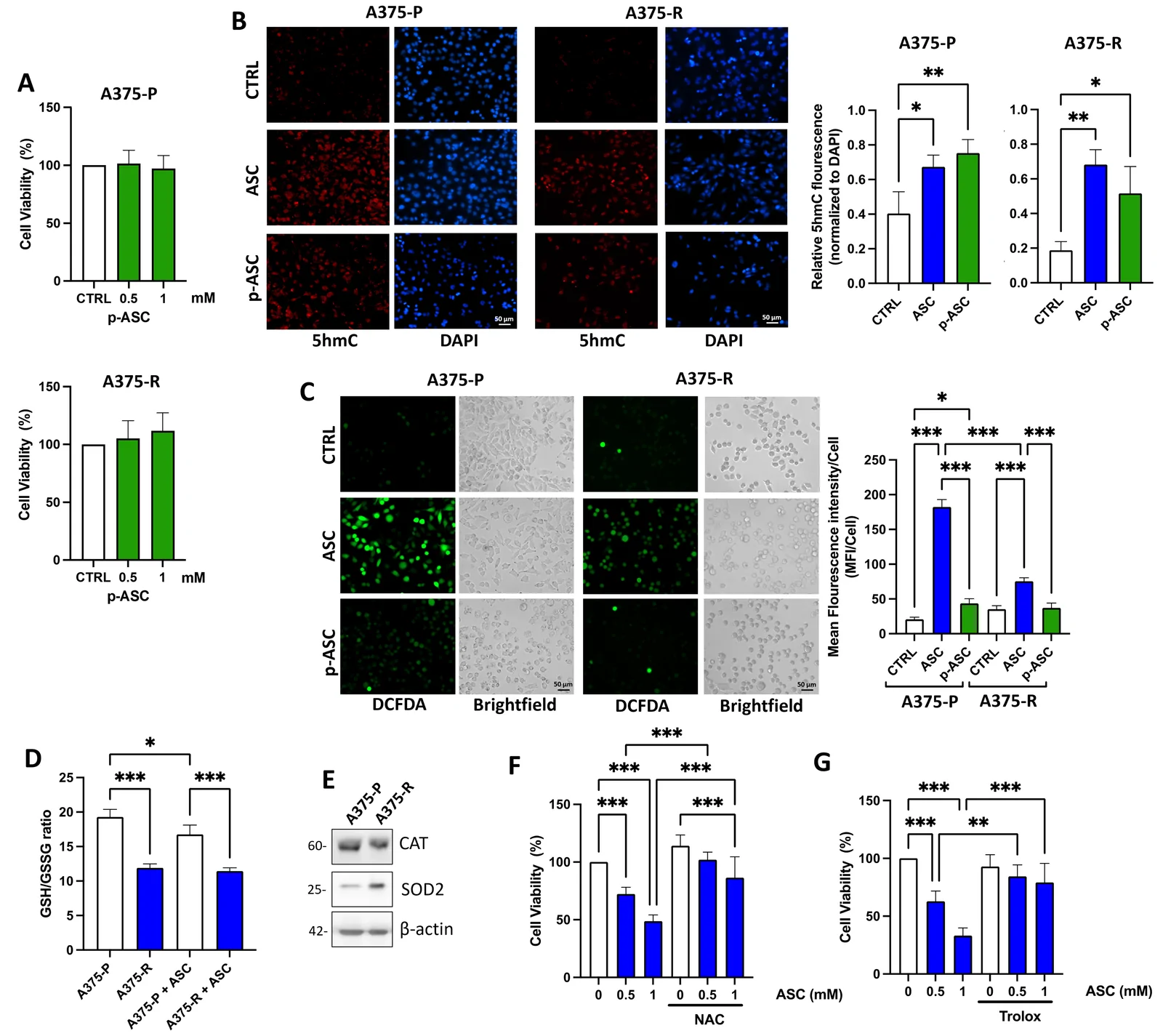
Analysis of ASC-induced ROS production and antioxidant response in A375 cells. (A) MTT assay of A375-P and A375-R treated with 0.5 and 1 mM p-ASC; (B) Representative images and fluorescence quantification of 5hmC levels in A375-P and A375-R treated with/without 0.5 mM ASC or p-ASC. Scale bar = 50 µm; (C) Representative images and fluorescence quantification of ROS levels measured by DCFDA in A375-P and A375-R treated with/without 0.5 mM ASC or p-ASC. Scale bar = 50 µm; (D) Fluorometric analysis of GSH/GSSG ratio in A375-P and A375-R treated with/without 0.5 mM ASC; (E) Western blot of SOD2 and CAT proteins in A375-P and A375-R; images are representative of three independent western blots; (F) NAC or (G) Trolox treatment in the presence of 0.5 or 1 mM ASC and analysis of cell viability by MTT assay in A375-R cells. Where indicated, CTRL refers to PBS used as the vehicle control for ASC or p-ASC. Statistical analysis in all bar graphs was performed by one-way ANOVA. ^*^P < 0.05; ^**^P < 0.01; ^***^P < 0.001. All experiments were performed in *n* = 3 independent biological replicates. ASC: L-ascorbic acid; ROS: reactive oxygen species; MTT: 3-[4,5-dimethylthiazol-2-yl]-2,5 diphenyl tetrazolium bromide; p-ASC: L-ascorbic acid 2-phosphate; 5hmC: 5-hydroxymethylcytosine; DCFDA: 2′,7′-dichlorofluorescein diacetate; SOD2: superoxide dismutase 2; CAT: catalase; NAC: N-acetylcysteine; PBS: phosphate-buffered saline; ANOVA: analysis of variance; DAPI: 4′,6-diamidino-2-phenylindole.

High-dose ASC is also known to promote ROS generation^[21,22]^. To clarify whether oxidative stress contributes to ASC-mediated cytotoxicity, we tested equimolar concentrations of p-ASC, an oxidation-resistant and more stable ASC derivative. Unexpectedly, p-ASC failed to induce cytotoxicity in either dabrafenib-sensitive or -resistant A375 cells [Figure 3A]. Despite this, p-ASC elevated 5hmC levels to an extent comparable to ASC [Figure 3B], indicating that TET activation alone does not account for ASC-induced cell death. We therefore hypothesized that the distinct biological effects of ASC and p-ASC stem from their different pro-oxidant properties. DCFDA analysis showed a significant increase in ROS following ASC treatment, whereas p-ASC was considerably less effective [Figure 3C]. Interestingly, the analysis of the glutathione antioxidant system showed a lower ratio of reduced (GSH) to oxidized (GSSG) forms - indicative of greater oxidative stress - in parental cells exposed to ASC compared with untreated samples, whereas no change was detected in A375-R [Figure 3D]. In addition, the analysis of the antioxidant enzymes superoxide dismutase 2 (SOD2) and catalase (CAT) revealed higher basal SOD2 expression in resistant cells, supporting their reduced sensitivity to ASC [Figure 3E]. Notably, by using the antioxidant compounds NAC [Figure 3F] or Trolox [Figure 3G] in combination with ASC, we observed that cell viability was completely recovered.

### ASC-induced cytotoxicity is associated with GLUT1 expression and is dependent on TET2 in A375 cells

**Figure 4:**
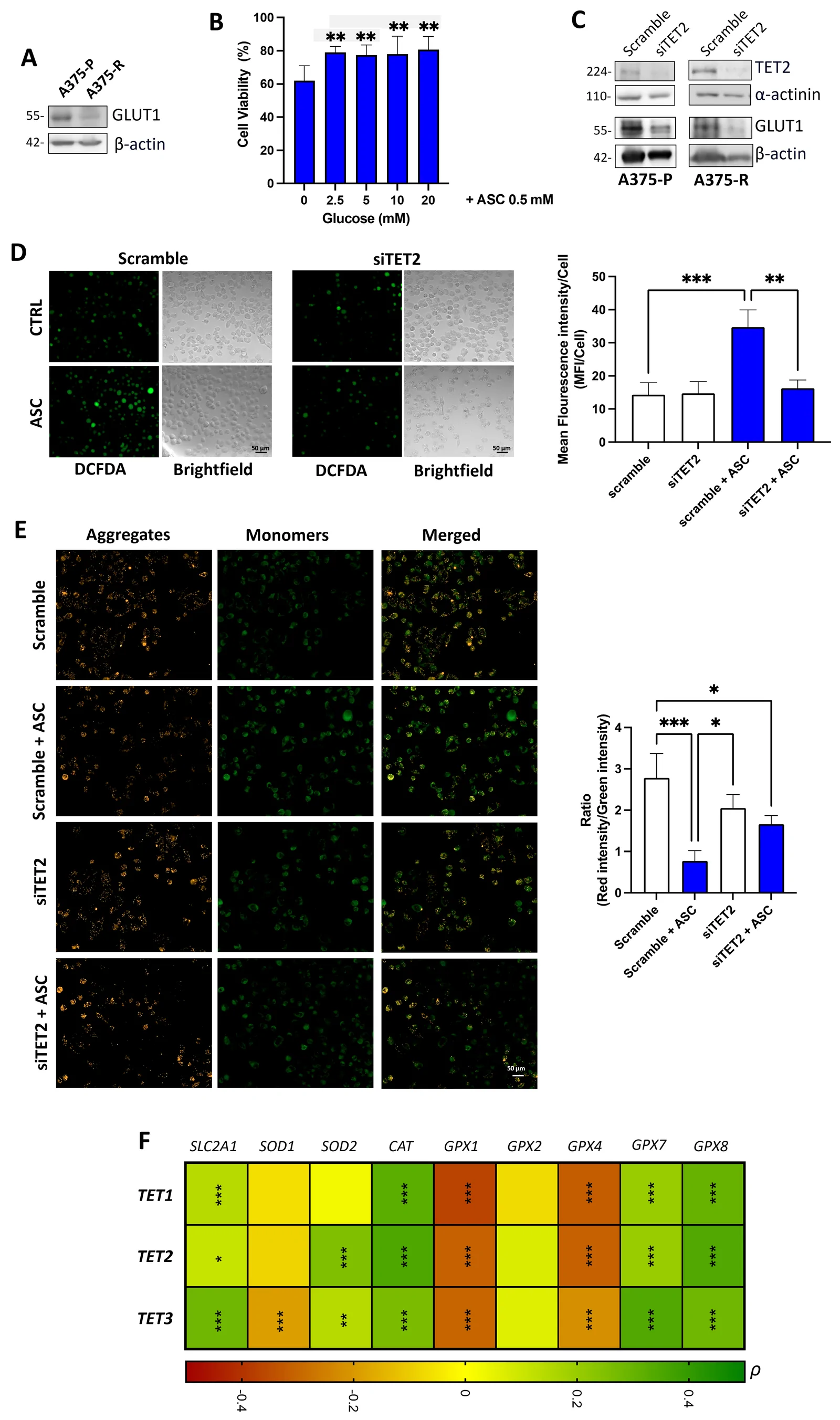
Analysis of TET2 and GLUT1 in the response of A375 cells to ASC. (A) Western blot of GLUT1 protein in A375-P and A375-R; images are representative of three independent western blots; (B) MTT assay of A375-R treated with 0.5 mM ASC in the presence of increasing concentrations of glucose; (C) Western blot of TET2 and GLUT1 in A375-P and A375-R silenced for *TET2*; images are representative of three independent western blots; (D) Representative images and fluorescence quantification of ROS levels measured by DCFDA in A375-R silenced for *TET2* and treated with/without 0.5 mM ASC; Scale bar = 50 µm; (E) Representative images and fluorescence quantification of mitochondrial polarization measured by JC-1 in A375-R silenced for *TET2* and treated with/without 0.5 mM ASC; Scale bar = 50 µm. Where indicated, CTRL refers to PBS used as the vehicle control for ASC. Statistical analysis in all bar graphs was performed by one-way ANOVA; (F) Spearman’s correlation analysis performed on melanoma samples from SKCM-TCGA dataset. ^*^P < 0.05; ^**^P < 0.01; ^***^P < 0.001. All experiments were performed in *n* = 3 independent biological replicates. TET: Ten-Eleven Translocation; ASC: L-ascorbic acid; MTT: 3-[4,5-dimethylthiazol-2-yl]-2,5 diphenyl tetrazolium bromide; ROS: reactive oxygen species; DCFDA: 2′,7′-dichlorofluorescein diacetate; PBS: phosphate-buffered saline; ANOVA: analysis of variance; SKCM: Skin Cutaneous Melanoma; TCGA: The Cancer Genome Atlas.

The mechanism underlying ROS production typically depends on the oxidation of high-dose ASC in the extracellular space and its subsequent reduction once inside the cell, which induces a redox imbalance^[22,23]^. Because the uptake of oxidized ASC relies on the GLUT1 transporter^[24]^, we examined its expression in dabrafenib-sensitive and -resistant cell lines. Interestingly, GLUT1 protein levels were lower in A375-R than in A375-P cells [Figure 4A]. Glucose transporter saturation induced by elevated glucose levels attenuated ASC cytotoxicity [Figure 4B], supporting the notion that ASC relies on GLUT-mediated uptake to enter cells. We next investigated whether GLUT1 protein levels are regulated by TET2 by performing gene-silencing experiments. These analyses revealed that GLUT1 protein levels were reduced following TET2 knockdown in both A375-P and A375-R cells [Figure 4C]. Finally, we demonstrated that TET2 silencing attenuates ASC-induced cytotoxicity by altering ROS generation. DCFDA fluorescence increased only in SCR-transfected cells upon ASC exposure, whereas ROS levels remained comparable to untreated controls in TET2-silenced cells, even after ASC treatment [Figure 4D]. Consistent with these findings, mitochondrial depolarization (low red-to-green JC1 ratio) in ASC-treated SCR cells is not evident following TET2 depletion [Figure 4E], further supporting a TET2-dependent mechanism underlying ASC-induced oxidative cytotoxicity. Finally, analysis of RNA-seq datasets from cutaneous melanoma human samples (SKCM-TCGA) showed that *TET2*, as well as *TET1* and *TET3*, positively correlates with genes encoding GLUT1 (i.e., *SLC2A1*) and the H_2_O_2_ detoxifying enzymes CAT and GSH peroxidase (GPX) 7 and 8 [Figure 4F].

### TET2 promotes GLUT1 expression, ASC-induced oxidative stress and cytotoxicity in M14 melanoma cells

**Figure 5:**
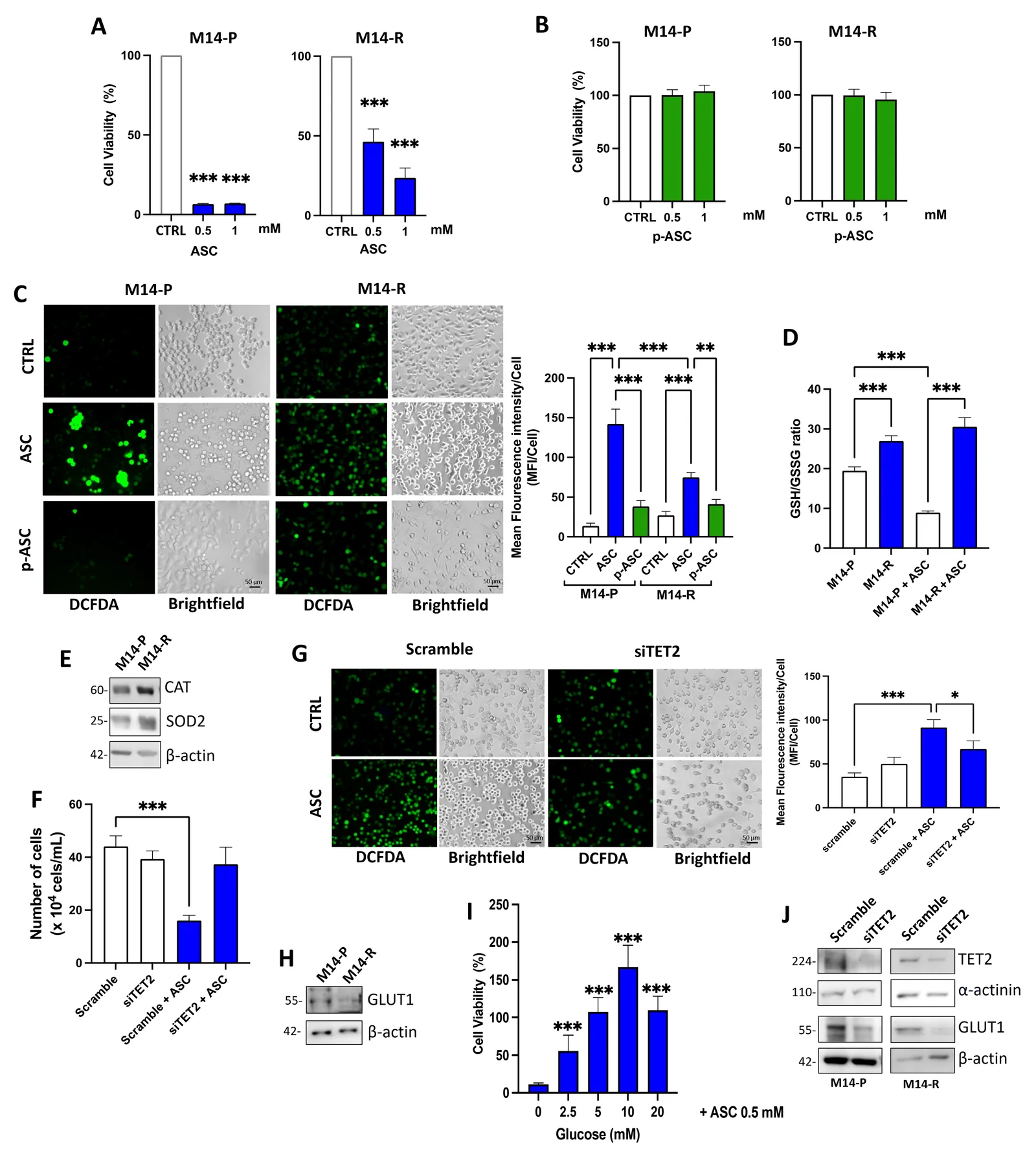
Analysis of ASC response mechanisms in M14 melanoma cells. MTT assay of M14-P and M14-R treated with (A) 0.5 and 1 mM ASC or (B) 0.5 and 1 mM p-ASC; (C) Representative images and fluorescence quantification of ROS levels measured by DCFDA in M14-P and M14-R treated with/without 0.5 mM ASC or p-ASC; Scale bar = 50 µm; (D) Fluorometric analysis of GSH/GSSG ratio in M14-P and M14-R treated with/without 0.5 mM ASC; (E) Western blot of SOD2 and CAT proteins in M14-P and M14-R; images are representative of three independent western blots; (F) Direct cell counting of M14-R cells silenced for *TET2* with/without 0.5 mM ASC; (G) Representative images and fluorescence quantification of ROS levels measured by DCFDA in M14-R silenced for *TET2* and treated with/without 0.5 mM ASC; Scale bar = 50 µm; (H) Western blot of GLUT1 proteins in M14-P and M14-R; images are representative of three independent western blots; (I) MTT assay of M14-R treated with 0.5 mM ASC in the presence of increasing concentrations of glucose; (J) Western blot of TET2 and GLUT1 in M14-P and M14-R silenced for *TET2*; images are representative of three independent western blots. Where indicated, CTRL refers to PBS used as the vehicle control for ASC and/or p-ASC. Statistical analysis in all bar graphs was performed by one-way ANOVA. ^*^P < 0.05; ^**^P < 0.01; ^***^P < 0.001. All experiments were performed in *n* = 3 independent biological replicates. ASC: L-ascorbic acid; MTT: 3-[4,5-dimethylthiazol-2-yl]-2,5 diphenyl tetrazolium bromide; p-ASC: L-ascorbic acid 2-phosphate; ROS: reactive oxygen species; DCFDA: 2′,7′-dichlorofluorescein diacetate; SOD2: superoxide dismutase 2; CAT: catalase; TET: Ten-Eleven Translocation; PBS: phosphate-buffered saline; ANOVA: analysis of variance.

To corroborate the results obtained in the A375 cell line, we repeated the key experiments in M14 cells. We confirmed that ASC is less cytotoxic in M14-R than in M14-P cells [Figure 5A], while neither cell line was affected by p-ASC [Figure 5B]. Although both compounds increased 5hmC levels in the M14 pair [Supplementary Figure 1A], the pro-oxidant effect of ASC was particularly pronounced in the parental line [Figure 5C], as reflected by a marked decrease in the GSH/GSSG ratio [Figure 5D]. M14-R also showed higher levels of SOD2 and CAT [Figure 5E]. TET2 silencing reversed the cytotoxic effects of ASC [Figure 5F], the ASC-induced increase in 5hmC levels [Supplementary Figure 1B], ROS generation [Figure 5G], and mitochondrial depolarization [Supplementary Figure 1C]. Consistent with observations in A375 cells, the parental M14 line expressed higher levels of GLUT1 than its resistant counterpart [Figure 5H]. Moreover, increasing glucose concentrations limited ASC-induced cytotoxicity [Figure 5I] and TET2 knockdown effectively downregulated GLUT1 protein levels [Figure 5J].

## DISCUSSION

Our data identify altered expression of the TET family enzymes as a feature of dabrafenib-resistant A375 and M14 cells and implicate TET2 in controlling the cytotoxic response to pharmacological ASC. Independent RNA-seq datasets from vemurafenib-resistant or encorafenib-exposed melanoma lines are consistent with such adaptive TET remodeling during BRAFi response. These findings dovetail with prior reports showing that reduced TET activity contributes to melanoma progression^[11,14]^ while also extending this epigenetic axis to the context of BRAFi resistance and redox-dependent cell killing. Accordingly, activation of TET activity by ASC has also been shown to synergize with other chemotherapeutic agents in sensitive and drug-resistant cells from different cancer types, including colon cancer^[25]^, chemoresistant BRCA1/2-deficient cells^[26]^ and arsenic trioxide-resistant acute promyelocytic leukemia^[27]^.

The literature on BRAFi resistance highlights extensive metabolic and redox rewiring in resistant cells, including increased mitochondrial dependence^[28]^, rewired sulfur metabolism^[29]^, and antioxidant buffering^[30]^. All these processes could intersect with Fe(II)/2-oxoglutarate-dependent activity of TET enzymes via altered cofactor and redox dysregulation. Consistently, the cofactor ASC increased 5hmC levels and reduced viability in BRAFi-sensitive A375 and M14 cells in a ROS-dependent manner, while resistance to dabrafenib coincided with reduced ASC sensitivity. Although we cannot exclude a potential contribution of TET1 and TET3 to the cytotoxic response induced by ASC, our data clearly identify TET2 as a key mediator of this process. Specifically, TET2 silencing abrogated ASC-induced 5hmC accumulation and cell death, establishing TET2 as necessary for the epigenetic component of the ASC response. The limited production of 5hmC observed following TET2 silencing further reinforces this conclusion and is consistent with the relevant biochemical role of ASC as a cofactor for TET2 activity^[20]^, whereby it sustains catalysis by maintaining iron in its reduced state at the active site. Consistently, multiple studies have shown that ASC boosts TET2 activity and 5hmC in cancer models^[31,32]^. However, equimolar p-ASC - an oxidation-resistant ASC derivative - raised 5hmC comparably to ASC but failed to trigger cytotoxicity, indicating that TET activation alone does not account for cell death. In line with this observation, low doses of ASC, which primarily exert antioxidant rather than pro-oxidant effects, are unlikely to induce cytotoxicity, as observed with p-ASC. Therefore, our data support a two-hit mechanism: (i) TET2-dependent epigenetic reprogramming that may license specific expression outputs; and (ii) pro-oxidant activity of high-dose ASC with subsequent intracellular redox collapse.

Mechanistically, the ROS-dependent cytotoxicity of pharmacological (high-dose) concentrations of ASC requires efficient cellular entry of the oxidized form of ASC, primarily via GLUT1^[33,34]^. Accordingly, the analysis of transporters of reduced ASC (SVCT1 and SVCT2) was not considered relevant in this setting. Our findings that (i) GLUT1 is higher in parental than in resistant cells; (ii) ASC lowers the GSH/GSSG ratio preferentially in parental cells and collapses mitochondrial potential; and (iii) antioxidants fully rescue viability, collectively argue that ASC uptake and glutathione consumption are central to ASC-mediated killing. These results are firmly grounded in the literature: GLUT1 transports oxidized ASC; once inside the cell, it is rapidly reduced back at the expense of intracellular reductants, depleting the glutathione pool and amplifying ROS^[21,33,35]^. Importantly, we find that TET2 knockdown reduces GLUT1 protein levels and blunts ASC-induced ROS, positioning TET2 upstream of a metabolic entry point for oxidized ASC. Although the molecular mechanism linking TET2 and GLUT1 was not investigated, TET2 may facilitate the susceptibility to pro-oxidant doses of ASC by coupling epigenetic state to redox pharmacology.

The attenuated ROS burst and preserved GSH/GSSG ratio in resistant cells after ASC exposure, together with their higher basal SOD2 (and in M14 catalase) levels, suggest that enhanced antioxidant defenses buffer the oxidative load imposed by ASC cycling. Elevated SOD2 has been reported as part of the adaptive response to BRAF/MEK inhibition, functionally contributing to the tolerant/resistant phenotype^[36]^. Our data recapitulate these relationships and provide a mechanistic explanation for the diminished efficacy of ASCs in BRAFi-resistant melanoma.

From a clinical and translational perspective, our results first underscore that “epigenetic restoration” by ASC (i.e., increasing 5hmC via TET2) is separable from and insufficient for killing in melanoma; achieving cytotoxicity requires concurrent pro-oxidant chemistry and adequate ASC influx. This helps reconcile several preclinical and clinical experiences with high-dose ASC across tumor types by highlighting context dependency on transporter expression and antioxidant capacity^[10,37]^. Therefore, actionable biomarkers for selecting tumors that may benefit from ASC-based strategies are TET2 abundance/function, GLUT1 levels, and redox buffering status (SOD2/CAT and glutathione metabolism). Notably, our analysis of SKCM-TCGA indicates positive correlations between TETs, SLC2A1, and peroxidase genes (CAT, GPX7/8), consistent with the coordinated epigenetic–metabolic program we observe experimentally.

A key limitation of this study is the lack of *in vivo* validation, including patient-derived models. The therapeutic relevance of our findings critically depends on the route of ASC administration, as pro-oxidant activity requires sufficiently high concentrations. Oral administration is tightly regulated and typically results in plasma levels of 100-200 μM, which are insufficient to reproduce the pro-oxidant effects observed *in vitro*. In contrast, intravenous administration reliably achieves millimolar concentrations (1-20 mM) necessary for anticancer activity^[10]^. Therefore, the effects described here are specifically aligned with intravenous ASC-based therapeutic strategies rather than dietary supplementation.

Another important limitation is the lack of data on the role of ASC during the development of BRAFi resistance, beyond its cytotoxic effects in established resistant cells. One may speculate that maintaining low ASC doses during the development of resistance could help preserve TET activity, thereby counteracting the epigenetic reprogramming associated with TET downregulation. However, the overall impact on the timing and nature of resistance remains uncertain and warrants direct investigation. If high doses of ASC were used in combination with dabrafenib, one could hypothesize enhanced cytotoxicity in parental cells through the additive or synergistic pro-oxidant effects of ASC and BRAF inhibition. This combined effect could lead to more effective tumor cell eradication or, at least, a significant reduction in the pool of surviving cells, thereby delaying the emergence of resistant clones. Consistently, the potential development of resistance to ASC itself cannot be excluded, as such mechanisms have already been reported in other malignancies, including pancreatic cancer^[38]^.

In conclusion, our results uncover a TET2-centered mechanism that couples the epigenetic state to oxidative stress–mediated killing by pharmacological concentrations of ASC in melanoma cells. Although ASC is a well-established activator of TET enzymatic activity, our findings extend this paradigm by identifying TET2 not merely as a downstream effector of ASC, but as a critical determinant of its intracellular efficacy. Specifically, we demonstrate that TET2 is required for ASC-mediated pro-oxidant cytotoxicity, acting as a permissive node that links ASC uptake and intracellular processing to the establishment of a pro-oxidant state.

Accordingly, rather than a unidirectional relationship in which ASC activates TET enzymes, our data support a bidirectional functional dependency in which TET2 also shapes the cellular response to ASC, including its cytotoxic effects. By linking TET2 to GLUT1 expression and ROS generation, this work defines a previously unrecognized regulatory axis integrating epigenetic state and redox vulnerability in BRAFi resistance, providing new mechanistic insight into ASC anticancer activity.

## DECLARATIONS

### Availability of data and materials

Data are available from the corresponding authors upon request.

### AI and AI-assisted tools statement

During the preparation of this manuscript, the AI tool Copilot (version 3.3.19, released 2025-11-10) was used solely for language editing. The tool did not influence the study design, data collection, analysis, interpretation, or the scientific content of the work. The refinement of the graphical abstract was performed by using Gemini AI (Gemini 3.5 Flash version). All authors take full responsibility for the accuracy, integrity, and final content of the manuscript.

### Copyright

© The Author(s) 2026
