# Supplementary material for: TET2 downregulation limits oxidative stress-induced cytotoxicity of high dose ascorbate in dabrafenib-resistant melanoma cells
Source: Cancer Drug Resist. 2026 Aug 3;9:27. doi: 10.20517/cdr.2026.42 (PMC13563456; doi:10.20517/cdr.2026.42)
Supplement: Supplementary file 1 [file cdr-9-27-SupplementaryMaterials.pdf]

Supplementary Materials

TET2 downregulation limits oxidative stress-induced cytotoxicity of high dose ascorbate in dabrafenib-resistant melanoma cells

Eleicy Nathaly Mendoza[1], Serena Castelli[2,3], Angela De Cristofaro[1], Enrico Desideri[4], Grazia Graziani[5], Pedro Miguel Lacal[6], Maria Rosa Ciriolo[1], Fabio Ciccarone[1]

[1]Department of Biology, University of Rome Tor Vergata, Rome 00157, Italy.

[2]Department for the Promotion of Human Science and Quality of Life, San Raffaele Open University, Rome 00166, Italy.

[3]IRCCS San Raffaele Roma, Rome 00166, Italy.

[4]Department of Life Sciences, Health and Health Professions, Link Campus University, Rome 00165, Italy.

[5]Department of Systems Medicine, University of Rome Tor Vergata, Rome 00133, Italy.

[6]Laboratory of Molecular Oncology, IDI-IRCCS, Rome 00167, Italy.

Correspondence to: Prof. Fabio Ciccarone, Department of Biology, University of Rome Tor Vergata, Rome 00157, Italy. E-mail: fabio.ciccarone@uniroma2.it

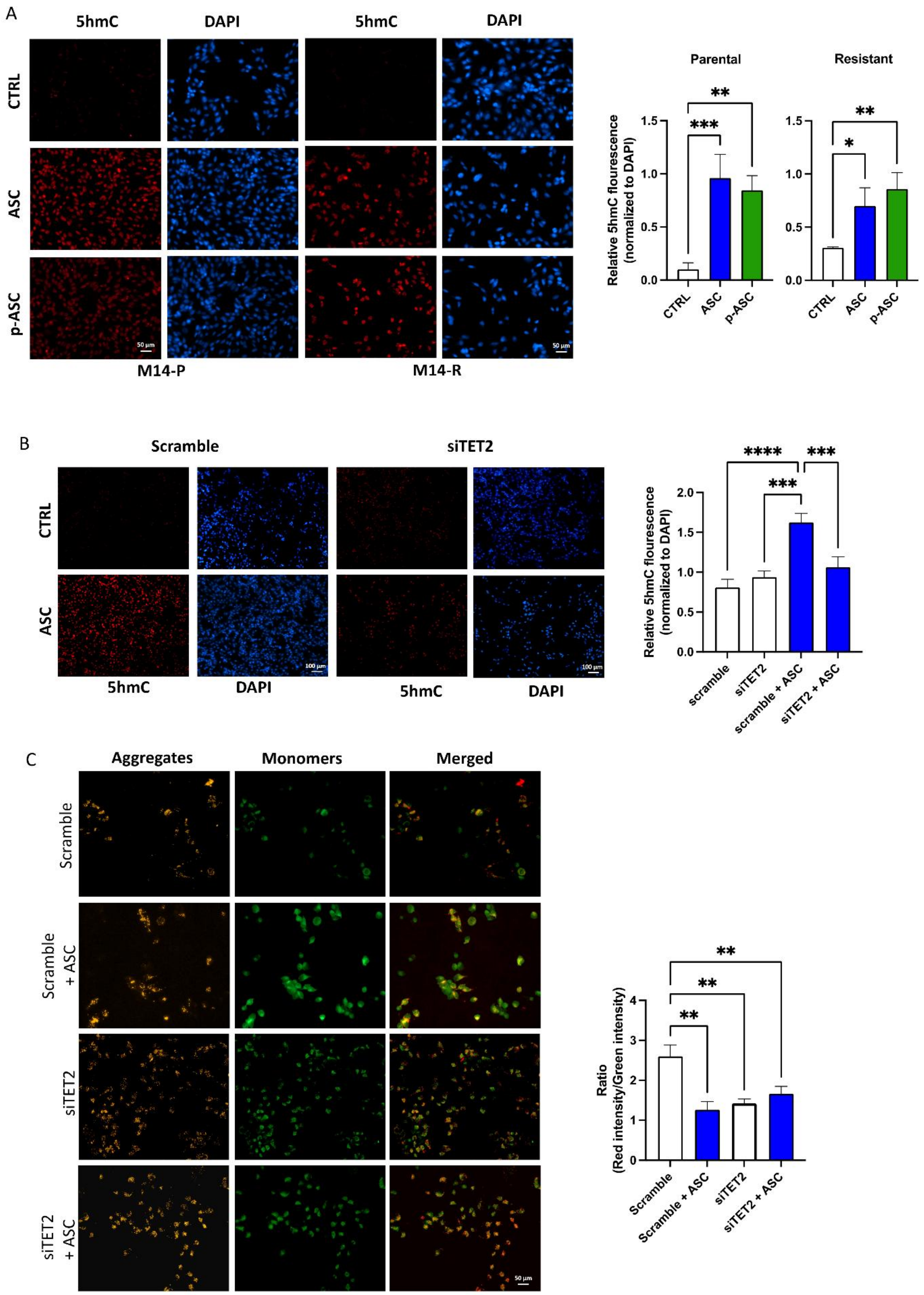

Supplementary Figure 1. Analysis of the pro-oxidant and epigenetic effects ASC and TET2 in M14 cells. A) Representative images and fluorescence quantification of 5hmC levels in M14-P and M14-R treated with/without 0.5 mM ASC or p-ASC; Scale bar = 50 µm. B) Representative images and fluorescence quantification of 5hmC levels in M14-R cells silenced for TET2 and treated with/without 0.5 mM ASC for 24 h; Scale bar = 100 µm. C) Representative images and fluorescence quantification of mitochondrial polarization measured by JC-1 in M14-R silenced for TET2 and treated with/without 0.5 mM ASC for 24 h; Scale bar = 50 µm. Statistical analysis in all bar graphs was performed by one-way ANOVA. $^{*}p < 0.05$; $^{**}p < 0.01$; $^{***}p < 0.001$; $^{****}p < 0.0001$.
